# Rural Residence and One-Person Households Are Associated with Diagnostic Delay in Pulmonary Tuberculosis in a Low-Incidence European Setting

**DOI:** 10.3390/tropicalmed11050120

**Published:** 2026-05-04

**Authors:** Tatjana Munko, Vesna Vukičević Lazarević, Jelena Barišić, Marina Perković, Tanja Vignjević

**Affiliations:** 1 Special Hospital for Pulmonary Diseases, Rockefeller’s Street, 10000 Zagreb, Croatia; tanja.munko@pulmologija.hr (T.M.); bjelena1@gmail.com (J.B.); mpti2007@gmail.com (M.P.); 2 Roche Limited Liability Company, 10000 Zagreb, Croatia; tanjavignjevic@yahoo.com

**Keywords:** pulmonary tuberculosis, diagnostic delay, rural population, one-person household, health services accessibility

## Abstract

Objectives: Diagnostic delay in pulmonary tuberculosis remains a significant barrier to effective disease control, even in low-incidence settings. This study aimed to identify factors associated with total delay and its components among adults with pulmonary tuberculosis in such a setting. Patients and methods: A retrospective observational study was conducted on adults with pulmonary tuberculosis treated at a tuberculosis care centre in Croatia. Total delay was defined as the interval between symptom onset and treatment initiation. Data were collected through structured patient interviews using a standardized questionnaire, medical record review, and routine tuberculosis notification forms from the national public health registry. Sociodemographic and clinical predictors were evaluated using multivariable linear and logistic regression analyses. Results: Among 116 participants, the median total delay was 85 days (interquartile range 48.5–155.3). Rural residence was the strongest independent predictor, with patients experiencing an 88% longer delay than urban residents (*p* = 0.006). Individuals living in one-person households had a 49% longer delay (*p* = 0.047). Absence of chest pain was associated with shorter delay (−38%, *p* = 0.032) and lower odds of extreme delay (odds ratio 0.39, *p* = 0.047). Retired status independently predicted prolonged health system delay (42.1 days longer) and treatment delay (3.4 days longer). Conclusion: Prolonged delay may become increasingly important in the context of population ageing and changing household structures. Targeted strategies focused on rural, retired, and people living in one-person households may improve the timeliness of tuberculosis detection in settings where declining incidence can reduce clinical suspicion.

## 1. Introduction

Pulmonary tuberculosis remains one of the leading infectious causes of death worldwide, despite being both preventable and curable [1,2]. Delayed diagnosis contributes significantly to ongoing transmission, increased disease severity, and poorer treatment outcomes [3].

Although the burden of tuberculosis is highest in low- and middle-incidence countries, diagnostic delay continues to be reported even in low-incidence settings [3]. In such settings, delayed diagnosis may reflect reduced clinical suspicion rather than limited healthcare access. Identifying factors associated with delayed diagnosis is therefore essential for improving tuberculosis control strategies [4,5]. Although Croatia is classified as a low-incidence country, demographic transitions such as population ageing, rise in one-person households, and migration are placing increasing strain on the healthcare system, potentially hindering timely disease recognition [6,7,8,9]. Several studies from European low-incidence countries, including Portugal, Italy, and the United Kingdom, have reported persistent patient- and health-system-related delays despite otherwise favourable tuberculosis epidemiology [10,11,12]. Evidence regarding determinants of diagnostic delay in such settings remains limited [13].

The aim of this study was to identify factors associated with patient- and health-system-related delays among individuals diagnosed with pulmonary tuberculosis in a low-incidence European setting. Tuberculosis-related delay is a multidimensional concept that includes total delay as well as patient-, health system-, diagnostic-, and treatment-related components, helping identify where barriers arise along the care pathway [3]. In this study, we additionally examined prolonged delay (total delay above the median) and extreme delay (patients in the highest quartile of total delay). We hypothesized that patients’ sociodemographic characteristics and clinical features contribute to prolonged total delay.

The primary objective was to identify independent predictors of total delay among adults with pulmonary tuberculosis. Secondary objectives included examining determinants of individual delay components (patient, health system, diagnostic, and treatment delay) and estimating the burden of prolonged and extreme delay. Understanding these factors could inform targeted interventions to reduce diagnostic delays and strengthen tuberculosis control in low-incidence settings.

## 2. Materials and Methods

### 2.1. Study Design, Setting, and Population

A retrospective observational study was conducted at the Department of Pulmonary Diseases and Tuberculosis, Varaždin General Hospital, Klenovnik, Croatia, from December 2019 to December 2022. The study included self-presented adults aged ≥18 years with microbiologically confirmed pulmonary tuberculosis, as evidenced by positive cultures for Mycobacterium tuberculosis, performed using both solid Löwenstein–Jensen media and the liquid Mycobacterium Growth Indicator Tube (MGIT) system (Becton, Dickinson and Company, Franklin Lakes, NJ, USA). In the majority of patients (*n* = 85, 73.3%), the diagnosis had already been established earlier by direct microbiological methods (molecular testing and/or smear positivity), whereas in the remaining patients (*n* = 31, 26.7%), culture provided the first microbiological confirmation.

Patients identified through contact tracing or diagnosed incidentally during evaluations for unrelated medical conditions (e.g., preoperative assessments or screening prior to biological therapy) were excluded to minimize detection bias.

### 2.2. Predictors of Delay

Variables selected a priori as potential predictors of diagnostic delay included patients’ sociodemographic characteristics and clinical variables. Sociodemographic factors comprised age (continuous), sex (male/female), place of birth (Croatia, other European country, non-European country), place of residence (urban, suburban, or rural), education level (less than primary, primary, secondary, or higher), and employment status (employed, unemployed/housewife, or retired). Household composition was categorized as single-person household (one individual living alone) or multi-person household (two or more household members), in accordance with United Nations demographic classifications [14]. Clinical variables encompassed the presence of comorbidities (smoking and alcohol use, diabetes mellitus, chronic kidney disease, chronic pulmonary disease, hematologic disorders, chronic heart disease, and receipt of biologic therapy), as well as presenting symptoms of tuberculosis (cachexia, cough, fever, weight loss, haemoptysis, loss of appetite, and night sweats).

### 2.3. Outcomes

The primary outcome was total delay (TotD), defined as the time interval (in days) between symptom onset and initiation of anti-tuberculosis treatment. To improve interpretability, prolonged delay (PrD), defined as total delay above the median, was additionally examined to identify a broader subgroup of patients experiencing delays longer than the typical cohort experience. Extreme delay (ED), defined as total delay at or above the 75th percentile, was used to identify the most delayed subgroup within the study population [15]. Secondary outcomes included patient delay (PD), defined as the time from symptom onset to first healthcare contact; health system delay (HSD), defined as the time from first healthcare contact to treatment initiation; diagnostic delay (DD), defined as the time from symptom onset to tuberculosis diagnosis; and treatment delay (TD), defined as the time from diagnosis to treatment initiation. Definitions were based on previously published tuberculosis delay frameworks [3].

### 2.4. Data Collection

Data were collected retrospectively from routinely available sources during the study period (December 2019 to December 2022), during which eligible consecutive patients were included until the predefined sample size required for statistical analysis was reached. These sources included hospital medical records, a standardized structured questionnaire routinely completed by patients at the time of hospital admission for tuberculosis treatment, and routine tuberculosis notification forms from the national public health registry. The admission questionnaire provided information on age, sex, place of residence, education level, employment status, household composition, place of birth, comorbidities, smoking and alcohol consumption, as well as the onset and type of presenting tuberculosis symptoms. Age, sex, comorbidities, and presenting symptoms were cross-checked using medical records and registry documentation. Data on microbiological findings and dates of first healthcare contact, tuberculosis diagnosis, and treatment initiation were obtained from medical records and registry documentation. Because the timing of symptom onset was based partly on patient self-report, recall bias cannot be excluded.

### 2.5. Sample Size and Study Power

Sample size was estimated using G*Power 3.1.9.7 for linear multiple regression (fixed model, R^2^ deviation from zero), assuming α = 0.05, power = 80%, 9 predictors of total diagnostic delay, and a medium effect size (f^2^ = 0.15), yielding a required sample size of approximately 114 participants. The predictors were selected a priori based on clinical relevance and prior evidence: age, sex, place of residence, foreign-born status, education level, household composition, employment status, presence of comorbidities, and tuberculosis presenting symptoms [4,5]. A total of 116 participants met the eligibility criteria and were included in the final analysis.

### 2.6. Ethical Approval

The study was conducted in accordance with the principles of the Declaration of Helsinki. Ethical approval was obtained from the institutional ethics committee prior to study initiation (Ethical approval number—02/1-91/101-2021). All participants received written and verbal information about the study and provided written informed consent before enrollment. Participant confidentiality was strictly maintained, and all data were anonymised prior to analysis.

### 2.7. Statistical Analysis

Descriptive statistics were used to summarize participant characteristics. Continuous variables are presented as means with standard deviations or medians with interquartile ranges, depending on data distribution, whereas categorical variables are reported as frequencies and percentages. No missing data were present for the variables included in the final analyses. Normality was assessed using the Shapiro–Wilk test. Group comparisons were conducted using the Mann–Whitney U test for continuous variables, and the chi-square test or Fisher’s exact test for categorical variables when comparing two groups. For comparisons involving three or more groups, the Kruskal–Wallis test was applied, with post hoc Dunn’s test. Owing to a right-skewed distribution, TotD was log-transformed prior to regression analysis. Multivariable linear regression analyses were performed to identify independent predictors of prolonged delay while adjusting for potential confounders. The adjusted percentage change was calculated in Microsoft Excel. All other analyses were performed using JASP (version 0.95.2, University of Amsterdam, Amsterdam, The Netherlands). Statistical significance was defined as a two-sided *p*-value < 0.05

## 3. Results

### 3.1. Sociodemographic and Clinical Variables

A total of 116 patients were included in the study. Sociodemographic characteristics of patients are presented in Table 1. Furthermore, Table 2 presents data on comorbidities, while Table 3 summarizes the presenting symptoms.

### 3.2. Primary Outcome

#### 3.2.1. Total Delay

The median TotD was 85 days (IQR 48.5–155.25). TotD differed significantly according to place of residence (H (2) = 9.07, *p* = 0.011). Patients living in rural areas experienced the longest delay, with a median of 119 days (IQR 75.25–219.8), whereas those residing in urban and suburban areas had shorter delays, with medians of 72 days (IQR 39.5–118.5) and 81 days (IQR 45.5–116.5), respectively.

A statistically significant but weak positive correlation was observed between age and TotD (Spearman’s ρ = 0.19, *p* = 0.042), suggesting that older patients experienced slightly longer delays. In a linear regression model with log-transformed TotD as the dependent variable, age was identified as a significant predictor (B = 0.015, SE = 0.006, β = 0.24, *p* = 0.009). Each additional year of age was associated with an approximately 1.5% increase in delay. The model explained 5% of the variance in TotD (adjusted R^2^ = 0.051).

For all other tested variables presented in Table 4, there was no significant difference in TotD.

Patients presenting with chest pain experienced significantly longer TotD than those without this symptom (median 124 days, IQR 80.25–189.5 vs. 76.5 days, IQR 44–122; U = 1.589, *p* = 0.022). There was no significant difference in patients presenting with other symptoms.

A multiple linear regression model including nine a priori selected variables was performed. The analysis initially used log-transformed values to address skewness; these results are presented in Table 5. Of the presenting symptoms, only chest pain was retained in the model, as it was the sole symptom significantly associated with TotD in univariable analyses.

Rural residence, absence of chest pain, and one-person household emerged as independent predictors. Patients living in rural areas experienced a 88.3% longer diagnostic delay than urban residents (B = 0.633, 95% CI 20.08% to 195.65%, *p* = 0.006). Individuals living in a one-person household had a 49.2% longer delay (B = 0.400, *p* = 0.047), whereas patients without chest pain experienced a 38.5% shorter delay than those presenting with chest pain (B = −0.486, 95% CI −41.14% to −4.11%, *p* = 0.032). No significant associations were observed for age, sex, education level, comorbidity status, employment status, or place of birth.

#### 3.2.2. Prolonged Delay

PrD occurred in 58 participants (50.0%). A multivariable logistic regression model was performed using the same nine prespecified predictor variables as in the primary TotD analysis. Rural residence was the only independent predictor of PrD. Compared with urban residents, individuals living in rural areas had 3.59-fold higher odds of PrD (OR = 3.59, *p* = 0.018). No other variables were significantly associated with PrD.

#### 3.2.3. Extreme Delay

ED (>153.8 days) occurred in 25.9% of participants. When all nine prespecified variables were entered into the multivariable logistic regression model, no statistically significant associations were identified. Therefore, a parsimonious model was constructed that included only variables previously associated with TotD in the linear regression analysis.

In the adjusted model, shown in Table 6, chest pain remained the only independent predictor of ED. Patients without chest pain had significantly lower odds of experiencing ED than those with chest pain (OR = 0.39, *p* = 0.047). Household composition and place of residence were not significantly associated with ED after adjustment.

### 3.3. Secondary Outcomes

The median HSD was 22 days (IQR 11.75–55), while the median PD was 52 days (IQR 21–104.5). The median DD was 80 days (IQR 42.5–152.5), whereas the median TD was 0 days (IQR 0–2).

Multivariable linear regression analyses were subsequently performed for all secondary delay outcomes using the nine prespecified variables. For PD, rural residence was the only factor significantly associated with longer delay, with rural patients experiencing an average increase of 61.7 days compared with urban residents (B = 61.670, 95% CI 13.670–109.730, *p* = 0.012).

Regarding HSD, retired status emerged as the sole significant predictor; retired patients had a 42.1-day longer delay than employed individuals (B = 42.148, 95% CI 0.871–83.426, *p* = 0.045). Similarly, rural residence was independently associated with DD, corresponding to an average increase of 68.5 days relative to urban residence (B = 68.488, 95% CI 14.858–122.279, *p* = 0.013).

For TD, both rural residence and retired employment status were significant predictors. Patients living in rural areas experienced a 2.6-day longer TD (B = 2.620, 95% CI 0.080–5.159, *p* = 0.043), while retired patients had a 3.4-day longer delay compared with employed individuals (B = 3.391, 95% CI 0.082–6.700, *p* = 0.045). No other variables were significantly associated with the secondary outcomes.

## 4. Discussion

The total delay observed in our study exceeded that reported in comparable European settings [10,11,12]. Furthermore, earlier Croatian data from 2006, when Croatia was still classified as an intermediate tuberculosis burden country, reported a median health system delay of 15 days, seven days shorter than that observed in our 2019–2022 cohort [16]. This pattern may reflect the low-incidence paradox, whereby declining tuberculosis rates reduce clinical suspicion and contribute to slower diagnostic pathways [11].

These findings are of clear public health importance. Prolonged delay in pulmonary tuberculosis may extend the period of infectiousness, increase opportunities for community transmission, and result in more advanced disease at presentation [17]. Longer delays have also been associated with poorer treatment outcomes, greater morbidity, and increased mortality risk [18,19]. Diagnostic pathways may also influence treatment timing. Most patients in our cohort received rapid microbiological confirmation, whereas a smaller proportion were confirmed only after culture results became available. Treatment initiation may have depended on the degree of early diagnostic certainty, which could contribute to variation in TotD.

Although women in some settings experience longer diagnostic delays than men, this pattern is not universal and often reflects social and structural barriers rather than biological differences [20,21]. In our study, no significant sex-related differences were observed, consistent with earlier Croatian data [22]. This may reflect the context of a high-income European setting with comparatively good healthcare accessibility and greater gender equity, where barriers to care-seeking between women and men may be less pronounced than in more resource-constrained settings.

Furthermore, this study identified rural residence as the strongest independent determinant of prolonged TotD and PrD among adults with pulmonary tuberculosis in a low-incidence European setting. Individuals living in one-person households also experienced longer TotD, while the absence of chest pain was associated with shorter TotD and lower odds of ED. PD constituted the largest component of TotD, whereas retired status predicted HSD. Together, these findings highlight the importance of social context and healthcare accessibility in shaping diagnostic timelines.

Rural residence was associated with a particularly strong increase in both total delay (TotD) and prolonged delay (PrD), with patients experiencing nearly double the total delay compared with urban residents and higher odds of delay exceeding the median threshold. Similar patterns were also observed for PD, DD, and TD. Geographic barriers, reduced availability of specialized services, and transportation limitations may contribute to postponed healthcare seeking and slower referral pathways [23]. Primary care providers in rural areas may also encounter tuberculosis less frequently, potentially lowering clinical suspicion and delaying diagnostic evaluation [13,24]. Similar patterns have been reported in both high- and low-incidence countries, suggesting that spatial inequalities remain a persistent challenge for tuberculosis control regardless of overall disease burden [24,25,26].

Individuals living in a one-person household experienced longer TotD, suggesting that living in a multi-person household may facilitate timely healthcare seeking, because another household member may notice persistent symptoms, encourage medical consultation, or provide practical support [27]. Persons living alone may underestimate symptom severity, postpone medical evaluation, or face practical barriers such as transportation or reduced informal caregiving [28]. Targeted outreach may therefore benefit individuals with limited social support [29,30]. Because tuberculosis is a communicable disease, the household context may also be epidemiologically relevant. Household transmission represents an important pathway of tuberculosis spread, and delayed diagnosis may prolong exposure of co-residents before treatment initiation. Thus, while multi-person households may facilitate earlier symptom recognition, they may also involve greater potential for household transmission when delays occur [31]. However, these interpretations should be made cautiously, as detailed data on household crowding, family structure, and social support were not available in the present study. Furthermore, the observational design precludes causal inference.

Retired status independently predicted prolonged HSD and TD. Although older adults often have more frequent contact with healthcare providers, tuberculosis symptoms in this population may be misattributed to existing chronic conditions, age-related physiological changes, or general functional decline [32]. Such diagnostic overshadowing can complicate clinical assessment and contribute to delayed investigation [33]. Furthermore, atypical symptom presentation is more common among older individuals, potentially lowering clinical suspicion for tuberculosis during initial encounters [34,35]. Consistent with these observations, increasing age was associated with longer total diagnostic delay in our cohort, although the effect size was modest. This finding suggests that age-related clinical complexity may subtly prolong the diagnostic pathway, even when healthcare contact is frequent [33].

A notable finding was the association between chest pain and prolonged TotD, while the absence of chest pain was associated with shorter delay and lower odds of ED. This finding should be interpreted cautiously. Chest pain is a non-specific symptom that may initially prompt evaluation for more common alternative diagnoses such as cardiovascular disease or malignancy, potentially delaying consideration of tuberculosis. In low-incidence settings, such diagnostic diversion may be particularly relevant [36,37]. Residual confounding cannot be excluded, as factors such as smoking history, immunosuppression, pain severity, or socioeconomic characteristics were not fully captured [38]. Reverse causality is also possible, whereby prolonged undiagnosed tuberculosis may itself lead to chest pain and cardiovascular involvement [39,40]. Therefore, chest pain may be better interpreted as a marker of disease severity rather than a direct cause of delay [39].

The predominance of PD suggests that barriers to early healthcare seeking remain a major contributor to diagnostic latency [25,41]. Limited symptom recognition, stigma, and underestimation of disease severity may discourage timely consultation, supporting the need for public health strategies that improve tuberculosis awareness, particularly in rural populations [42]. Community-based educational strategies and targeted outreach could therefore represent effective complements to healthcare system improvements [29,43].

Our findings also reflect the “low-incidence paradox” whereby reduced exposure to tuberculosis may lower clinical suspicion during early encounters [11,44]. Sustained clinician awareness and guideline-based evaluation of persistent respiratory symptoms remain essential despite declining incidence [45].

This study has several notable strengths. The inclusion of microbiologically confirmed cases minimized diagnostic misclassification, while the use of predefined predictors reduced the risk of data-driven model construction. Furthermore, the simultaneous evaluation of TotD and its individual components enabled a more nuanced understanding of where delays occur along the diagnostic pathway. The application of multivariable analyses allowed adjustment for potential confounders and strengthened the validity of the observed associations.

Several limitations warrant consideration. Although the single-centre design may limit generalizability, tuberculosis care in Croatia is highly centralized, and the low national incidence (185, 158, and 173 new culture-positive cases in 2020, 2021, and 2022, respectively) enabled the inclusion of a large proportion of the target population [46]. Specifically, the study cohort of 116 participants represents approximately 22.4% of all newly diagnosed culture-positive cases during the three-year study period, supporting the representativeness of the findings despite the single-center design. However, while the sample size was sufficient for the planned regression analyses, smaller effect sizes may have gone undetected. Residual confounding cannot be excluded, and the observational design precludes causal inference. The timing of symptom onset was based partly on patient self-report, introducing the possibility of recall bias. Inaccurate recall of symptom onset may have affected estimates of total delay and its individual components, although data were collected using a standardized admission questionnaire. Furthermore, because prolonged and extreme delays were defined using cohort-specific distribution thresholds, direct comparison with studies applying fixed clinical cut-offs should be interpreted cautiously.

These findings have important implications for tuberculosis control in low-incidence settings. Improving healthcare access in rural areas, particularly for retired individuals living alone, alongside strengthening primary care recognition and public awareness of early symptoms, may reduce diagnostic delays. Enhancing standardized evaluation of persistent respiratory complaints through clear diagnostic algorithms and timely referral pathways could further improve case detection. Targeted awareness campaigns, digital triage tools in primary care, and mobile outreach services for geographically remote populations may also support earlier diagnosis and help limit ongoing community transmission [6].

## 5. Conclusions

The TotD is primarily influenced by geographic and social determinants. With population ageing and the rise in one-person households, targeted strategies that support early recognition and enhance clinical awareness will be essential for improving timely detection. As tuberculosis incidence declines, maintaining clinical vigilance will be essential to prevent diagnostic delay from becoming an unintended consequence of epidemiological success.

## Figures and Tables

**Table 1:** Sociodemographic characteristics of 116 adults with pulmonary tuberculosis diagnosed and treated between December 2019 and December 2022.

Characteristics		Total (*n* = 116)
Age (years), median (IQR)		53 (40.25–64)
Sex (*n*, %)	Male	85 (73.3%)
	Female	31 (26.7%)
Place of residence (*n*, %)	Urban	55 (47.4%)
	Suburban	27 (23.3%)
	Rural	34 (29.3%)
Education level (*n*, %)	Less than primary	9 (7.8%)
	Primary	65 (56%)
	Secondary or higher	42 (36.2%)
Employment status (*n*, %)	Employed	63 (54.3%)
	Unemployed Retired	22 (19%) 31 (26.7%)
Household composition (*n*, %)	One-person household Multi-person household	61 (52.6%) 55 (47.4%)
Place of birth (*n*, %)	Croatia	87 (75%)
	European country	26 (22.4%)
	Non-European country	3 (2.6%)
Any comorbidity (*n*, %)		44 (37.9%)
Smoking (*n*, %)		58 (50%)
Alcohol consumption (*n*, %)		57 (49.1%)

Values are presented as median (interquartile range) for continuous variables and number (percentage) for categorical variables. Comorbidities include diabetes mellitus, chronic kidney disease, chronic pulmonary disease, hematologic disorders, chronic heart disease, and receipt of biologic therapy.

**Table 2:** Comorbidities among 116 adults with pulmonary tuberculosis diagnosed and treated between December 2019 and December 2022.

Comorbidity	Total (*n* = 44)
Chronic lung disease (*n*, %)	20 (45.45%)
Diabetes mellitus (*n*, %)	10 (22.72%)
Chronic kidney disease (*n*, %)	2 (4.54%)
Hematologic disorders (*n*, %)	3 (6.81%)
Chronic heart disease (*n*, %)	4 (9.09%)
Receipt of biologic therapy (*n*, %)	5 (11.36%)

*n* = 44 represents participants with at least one recorded comorbidity. Percentages are calculated within this subgroup.

**Table 3:** Presenting symptoms among 116 adults with pulmonary tuberculosis diagnosed and treated between December 2019 and December 2022.

Symptom	Total (*n* = 116)
Cachexia (*n*, %)	17 (14.7%)
Cough (*n*, %)	111 (95.7%)
Increased body temperature (*n*, %)	54 (46.6%)
Loss of weight (*n*, %)	79 (68.1%)
Haemoptysis (*n*, %)	21 (18.1%)
Chest pain (*n*, %)	28 (24.1%)
Loss of appetite (*n*, %)	76 (65.5%)
Night sweats (*n*, %)	54 (46.6%)

**Table 4:** Univariable associations between participant characteristics and total delay (TotD) among adults with pulmonary tuberculosis (*n* = 116).

Characteristics		Median (IQR)	Test Statistic	*p*-Value
Sex	Male Female	79 (48–153) 90 (53–141.5)	U = 1.261	*p* = 0.727
Education level	Less than primary Primary Secondary or higher	81 (44–103) 95 (52–162) 78.5 (45.5–143.3)	H (2) = 1.041	*p* = 0.594
Employment status	Employed Unemployed Retired	74 (45.5–147) 85 (62.5–116.3) 112 (55.5–280.5)	H (2) = 4.65	*p* = 0.098
Household composition	One-person Multi-person	90 (58–156) 81 (38–133.5)	U = 1.448	*p* = 0.204
Place of birth	Croatia European country Non-European country	90 (51–154.5) 95.5 (52.75–168.5) 60 (52–67)	H (2) = 1.506	*p* = 0.471
Any comorbidity	Yes No	90 (53.5–186.5) 78.5 (47–146.5)	U = 1.404	*p* = 0.299
Comorbidity type	Chronic lung disease Diabetes mellitus Chronic kidney disease Hematologic disorders Chronic heart disease Receipt of biologic therapy	79.5 (43.75–120) 75.5 (42.25–142.8) 354.5 (309.3–399.8) 91 (80.5–177) 241 (161.8–328.8) 105 (81–112)	H (5) = 10.15	*p* = 0.103
Smoking	Yes No	78.5 (47.75–149.5) 89.5 (52.5–160.5)	U = 1.676	*p* = 0.976
Alcohol consumption	Yes No	78 (47–148) 91 (54–164)	U = 1.575	*p* = 0.556

Data are presented as categories compared for differences in total delay (TotD). U = Mann–Whitney U test; H = Kruskal–Wallis test statistic. *p*-values are two-sided.

**Table 5:** Multivariable linear regression analysis of factors associated with total delay (log-transformed) among 116 adults with pulmonary tuberculosis.

Predictors of TotD	B	% Change	95% CI	*p*-Value
Age	0.011	1.11	−0.6 to 2.74	0.223
Suburban residence	0.037	3.77	−34.82 to 65.04	0.876
Rural residence	0.633	88.33	20.08 to 195.65	0.006
Primary education	0.414	51.29	−34.82 to 202.53	0.239
Secondary education or higher	0.038	3.87	20.08 to 117.49	0.920
Any comorbidity	0.159	17.23	−24.35 to 77.71	0.451
Born in other European country	0.320	37.71	−50.39 to 121.22	0.184
Born outside Europe	0.534	70.57	−22.6 to 478.34	0.387
One-person household	0.400	49.18	−14.27 to 121.22	0.047
Female sex	0.073	7.57	−49.69 to 66.20	0.741
Unemployed	−0.018	−1.78	0.5 to 63.72	0.944
Retired	0.190	20.92	−30.44 to 117.49	0.523
Chest pain absent	−0.486	−38.49	−41.14 to −4.11	0.032

B = unstandardized regression coefficient. Percent change was calculated as (e^B − 1) × 100. Reference categories were urban residence, less than primary education, born in Croatia, multi-person household, male sex, employed, and presence of chest pain. TotD = total diagnostic delay, %change = adjusted percent change.

**Table 6:** Multivariable logistic regression analysis of factors associated with extreme delay among 116 adults with pulmonary tuberculosis.

Predictors of Extreme Delay	Adjusted OR	95% CI	*p*-Value
Suburban residence	0.680	0.209–2.212	0.410
Rural residence	1.798	0.672–4.808	0.243
One-person household	1.144	0.470–2.780	0.767
Chest pain absent	0.385	0.150–0.987	0.047

OR = odds ratio; CI = confidence interval. Extreme diagnostic delay was defined as total diagnostic delay equal to or exceeding the 75th percentile of the distribution. Variables included in the model were selected based on prior significance in the linear regression analysis. Reference categories were urban residence, multi-person household, and presence of chest pain.

## Data Availability

The raw data supporting the conclusions of this article will be made available by the authors on request.

## Abbreviations

The following abbreviations are used in this manuscript:

TB	Tuberculosis
TotD	Total delay
PrD	Prolonged Delay
ED	Extreme delay
PD	Patient delay
HSD	Health system delay
DD	Diagnostic delay
TD	Treatment delay
IQR	Interquartile range
OR	Odds ratio
CI	Confidence interval
MGIT	Mycobacterium Growth Indicator Tube
LJ	Löwenstein–Jensen medium
